# Substituted 2-Phenacylbenzoxazole Difluoroboranes: Synthesis, Structure and Properties

**DOI:** 10.3390/molecules25225420

**Published:** 2020-11-19

**Authors:** Agnieszka Skotnicka, Przemysław Czeleń

**Affiliations:** 1 Faculty of Chemical Technology and Engineering, UTP University of Science and Technology, Seminaryjna 3, 85-326 Bydgoszcz, Poland; 2 Department of Physical Chemistry, Collegium Medicum, N. Copernicus University, Kurpińskiego 5, 85-950 Bydgoszcz, Poland; przemekcz@cm.umk.pl

**Keywords:** difluoroboranes, substituent effect, synthesis, spectroscopic properties

## Abstract

Novel fluorescent dyes such as benzoxazole-boron complexes, bearing β-ketoiminate ligands, have been synthesized and characterized with a focus on the influence of a substituent on the basic photophysical properties. ^1^H, ^11^B, ^13^C, ^15^N, and ^19^F nuclear magnetic resonance (NMR) spectra of substituted 2-phenacylbenzoxazole difluoroboranes have been recorded and discussed. It is worth mentioning that a high correlation coefficient was found between ^15^N-NMR parameters and substituent constants. The photophysical properties of these new dyes have been investigated by fluorescence and ultraviolet-visible (UV-Vis) absorption spectroscopy. The geometry optimization, vibrational spectra, and the HOMO and LUMO energies were calculated based on density functional theory with the use of the B3LYP functional and 6-311++G(d,p) basis set.

## 1. Introduction

Boron dipyrromethene (BODIPY) dyes display excellent photophysical and optoelectronic properties, such as high photostability, a large absorption coefficient in the visible and near-infrared (IR) ranges, excellent chemical stability, high fluorescence quantum yields, relatively long excited-state lifetime, good solubility in organic solvents, relative insensitivity to environmental conditions, and good biocompatibility [1,2,3]. Moreover, the BODIPY chromophore is a versatile platform for the construction of fluorescent probes for bioimaging and biosensing applications [4,5,6]. In addition, novel photoinitiators based on the BODIPY are widely used for efficient cationic polymerization of epoxy, epoxy-silicone, and vinyl ether monomers [7,8].

There are mainly three types of these fluorine-boron complexes, classified as *N*,*N* bidentate, *O*,*O* bidentate, and *N*,*O* bidentate compounds. The typical BODIPY core contains two pyrrole units and its structure is usually symmetric. The compounds studied now contain NBF_2_O moiety. They carry an unsymmetrically chelated BF_2_ moiety. It was found that some difluoroboron β-ketoiminate boron complexes bearing benzoxazole gave strong emissions in solid states on account of aggregation-induced emission (AIE). The reversible mechanofluorochromism was due to the transformation between crystalline and amorphous states [9,10].

**Scheme 1:**
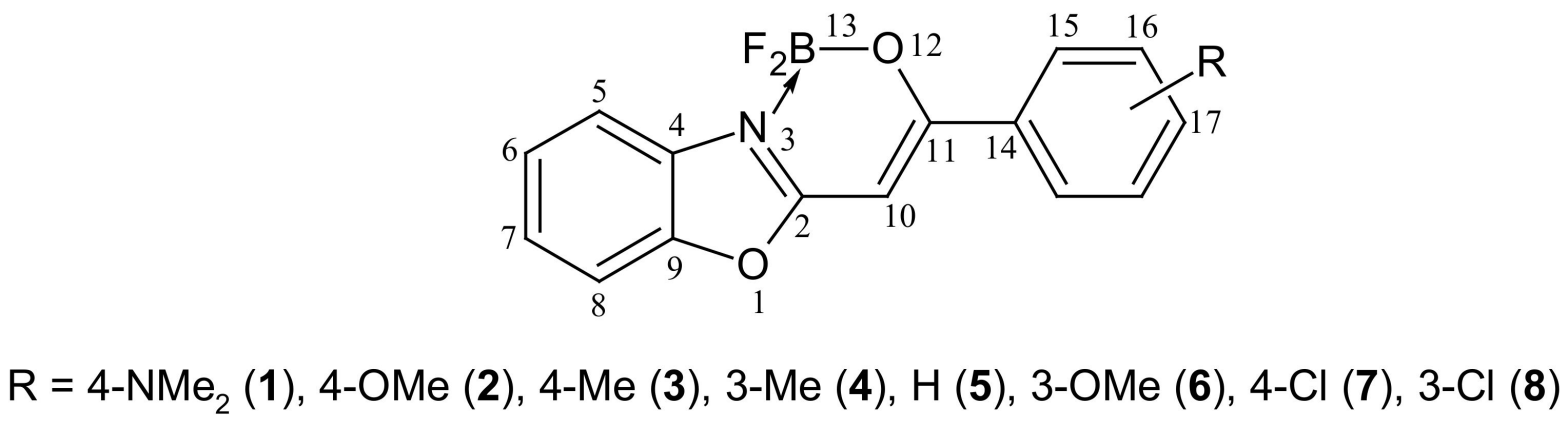
Structure and atom numbering in **1**–**8**.

In our previous published work, we have investigated the influence of heteroatom (X = O, S, and NMe) substitution in a five-membered heterocyclic ring on the photophysical properties of novel BF_2_ complexes containing or not a dimethylamino group (R = NMe_2_) [11]. It was shown that the fluorescence yields of the dimethylamino derivatives were much larger (up to one order of magnitude) than those of the corresponding unsubstituted compounds. Therefore, using the electron-donating or electron-accepting substituents, the electronic properties of the compounds may be tuned [12,13]. This study provides a report on the basic photophysical properties of a recently synthesized series of substituted 2-phenacylbenzoxazole difluoroboranes (Scheme 1) with a view towards the effect of substitution on their properties.

## 2. Results and Discussion

### 2.1. Synthesis and NMR Data

**Scheme 2:**
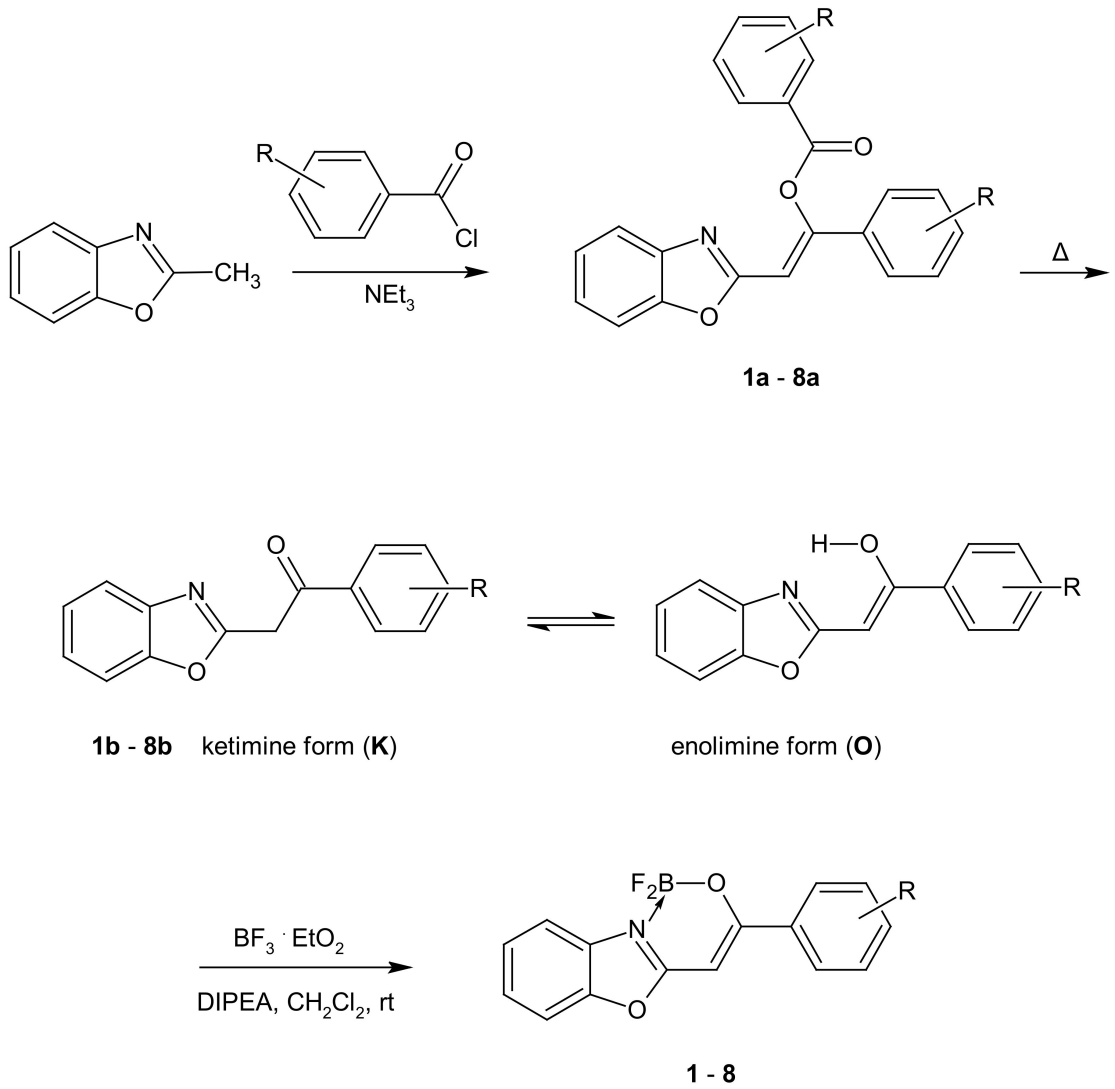
The schematic representation of the synthesis of 2-phencylbenzoxazole difluoroboranes where R = 4-NMe_2_ (**1**), 4-OMe (**2**), 4-Me (**3**), 3-Me (**4**), H (**5**), 3-OMe (**6**), 4-Cl (**7**), 3-Cl (**8**).

Treatment of 2-methylbenzoxazole with benzoyl chloride and triethylamine causes that the 2-methyl group undergoes double benzoylation, i.e., it is transformed to –CH=C(Ph)–COOPh. Non purified intermediate products **1a**–**8a** were always used in the subsequent synthetic step. By refluxing their morpholine solutions, **1a**–**8a** can be easily transformed into 2-phenacylbenzoxazoles **1b**–**8b** [14]. In solution, compounds **1b**–**8b** exist in two tautomeric forms, ketimine form (**K**) and enolimine form (**O**), through keto-enol tautomerization. The ketimine tautomer structure was confirmed by the methylene signals observed at the range of 4.54–4.67 ppm (s, 2H) in their ^1^H-NMR spectra. Enolimine tautomers were also confirmed by the ^1^H-NMR signals at 6.05–6.43 ppm (s, 1H, C=CH) and 12.4–12.8 ppm (s, 1H, OH) [15]. The tautomeric mixture **1b**–**8b** was then allowed to react with the boron trifluoride ethyl etherate in the presence of *N*-ethyldiisopropylamine in dichloromethane to give the corresponding BF_2_ complex **1**–**8** (Scheme 2).

The ^1^H, ^11^B, ^13^C, ^15^N, and ^19^F-NMR spectra that confirm the structures of **1**–**8** are maintained in solution. NMR chemical shifts of characteristic nuclei are collected in Table 1.

**Figure 1:**
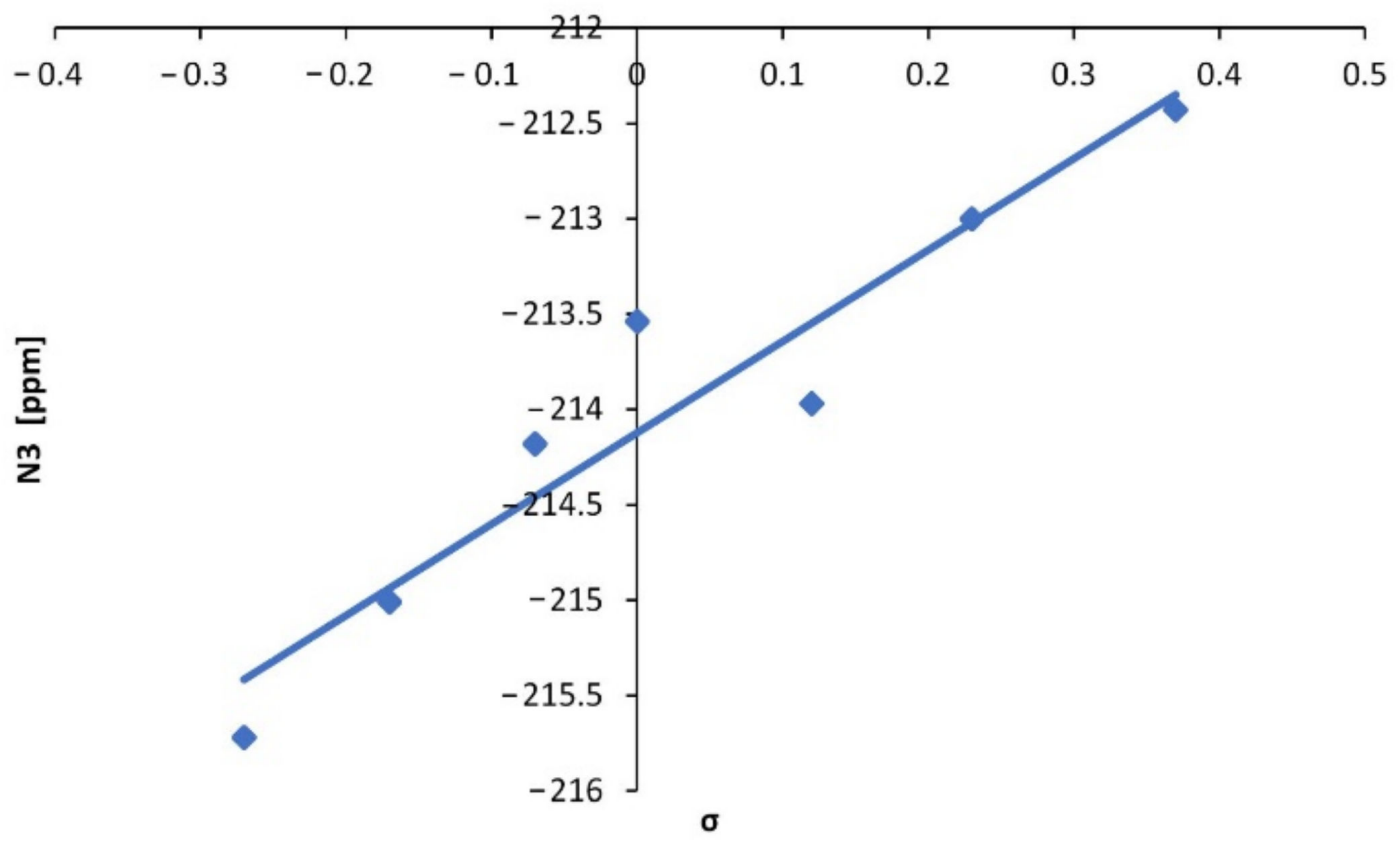
A plot of N3 chemical shift [ppm] vs. Hammett constant for **1**–**8**.

The ^1^H-NMR signal of H10 of 2-phenacylbenzoxazole difluoroboranes **1**–**8** can be seen at δ = 6.37–6.81 ppm in CDCl_3_, which is a singlet. The presence of a boron atom is confirmed by signals at δ = 1.79–1.85 ppm (triplet). The resonance of F13 at δ = −134.14 to −135.59 ppm shows as a quartet (two fluorine atoms). Analogously Kubota et al. [16] observed signals at −134.6 ppm (q, 2F) for boron complex of 2-methylbenzothiazole. N3 in the ^15^N-NMR spectra of 2-phenacylbenzoxazole difluoroboranes **1**–**8** resonates in the ranges of δ = −212.43 to −215.72 ppm. These signals were correlated to the Hammett substituent constant σ [17]. It was observed that with increasing electron-withdrawing ability of substituent, the ^15^N-NMR signal experienced a continuous upfield shift, which was indicated by a linear dependence of the chemical shift against σ (δ(15N) = 4.79 σ–214.12, R^2^ = 0.909), Figure 1. ^1^H NMR and ^13^C NMR spectra of **1**–**8** are available in Supplementary Materials.

### 2.2. Spectroscopic Properties

**Figure 2:**
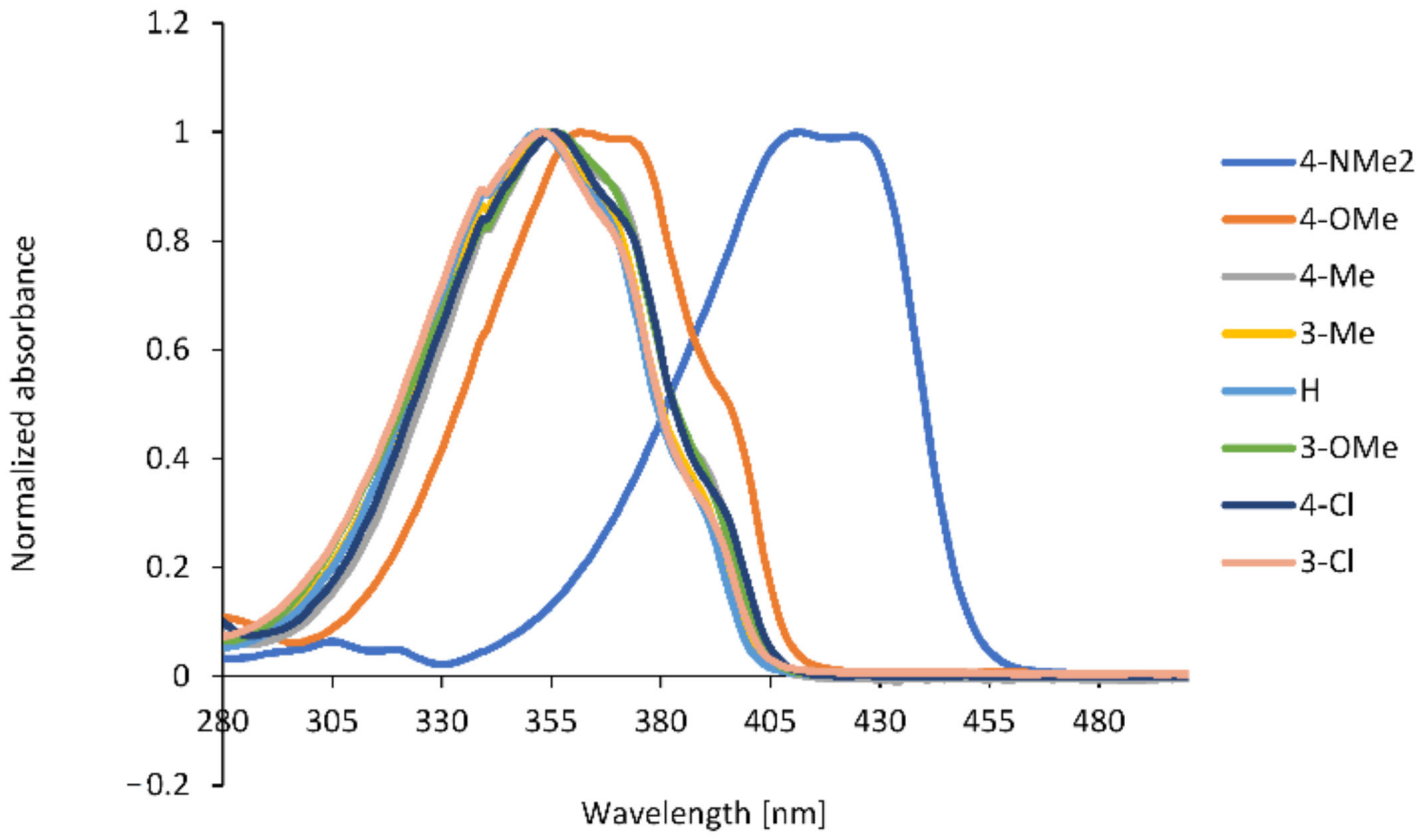
Normalized absorption spectra of **1**–**8** in chloroform (values were converted to have the maximum normalized at 1).

The measured absorption spectra of all eight compounds obtained in chloroform are given in Figure 2, and the associated data are presented in Table 2. Chloroform is a known solvent to prevent boron-ligand dissociation, exciplex formation, or the photochemical reactions possible in solvents containing Lewis bases, aromatic rings, or double bonds [18].

As shown in Figure 2, 2-phenacylbenzoxazole difluoroboranes **1**–**8** have an intense, wide absorption band in the 300–410 nm region, which is attributed to the π → π* transition but also associated with n → π* photochemically forbidden transition. The position of the absorption band depends on the dye structure. Absorption at λ_max_ was found to progressively shift to longer wavelength upon substituting unsubstituted complex **5** by weaker electron-withdrawing (Cl) and then electron-releasing (4-Me, 4-OMe, and 4-NMe_2_) substituents. It must be highlighted that the electron pulling effect from the boron atom created a strong electronic current in the six-membered ring such as B-O-C-C-C-N, which is responsible for the higher % of bathochromic in compound **2** in chloroform. A considerable red-shift of the absorption band was observed for compound **1** (4-NMe_2_). The 4-NMe_2_ substituent causes a 58 nm red shift in absorption relative to the parent compound **5**. The molar absorption coefficient (ε) of **1** (42,500) was significantly higher than for other complexes (Table 2). These red-shifts of the absorption maxima position along with the high molar absorption coefficients indicate that the transitions of the R = NMe_2_ derivative have a π → π* nature associated with a significant charge transfer (CT) [11].

**Figure 3:**
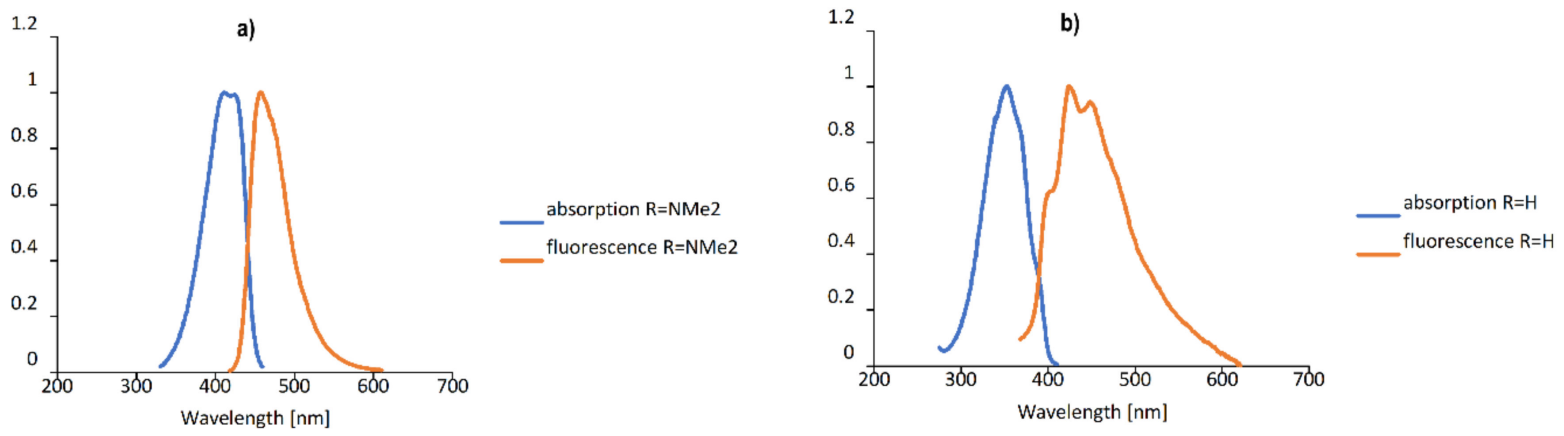
The normalized UV-Vis absorption (**a**) fluorescence and (**b**) spectra of **1** and **5** in CHCl_3_.

The changes in Stokes shifts (Figure 3) and the molar absorption coefficients related to the nature of the substituent in 2-phenacyl moiety are also reflected in changes in the fluorescence quantum yields. As can be seen in Table 2, the measurements of the fluorescence quantum yields of the investigated dyes remain very small. Only derivatives with electron-releasing groups exhibit fluorescence quantum yields significantly higher (up to one order of magnitude for **1**). 

**Figure 4:**
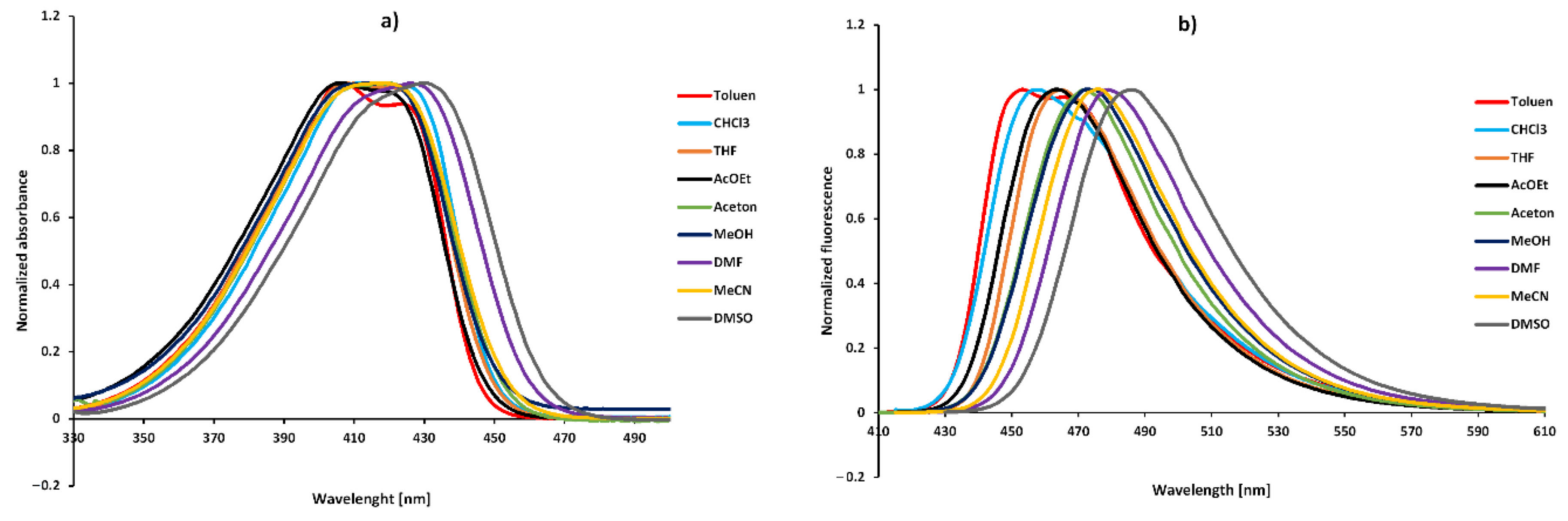
(**a**) Normalized absorption (**a**) and fluorescence (**b**) spectra of 2-(4-dimethylamino)benzoylbenzoxazole difluoroborane (**1**) in solvents of different polarity.

From the data presented in Table 3 and Figure 4, it is seen that the polar protic solvents such as methanol, dimethylformamide, acetonitryle, and dimethyl sulfoxide cause a bathochromic shift of the absorption band. The largest shift of the absorption bands towards longer wavelengths is observed for the most protic solvent, which is dimethyl sulfoxide. It is clear from Table 3 that the Stokes shift increases with varying solvent polarity. With an increase in solvent polarity, the magnitude of the Stokes shift varies from 2449 cm^−1^ to 3043 cm^−1^ for compound **1** (4-NMe_2_), and from 4530 cm^−1^ to 6464 cm^−1^ for other derivatives.

### 2.3. Solvatochromism

To verify the effect of solvent polarity, Stokes shifts (Δ*ν*) of **1**–**8** in a variety of solvents were plotted against the solvent polarity parameter Δ*f* (*ε*, *n*) with the general form of the Lippert-Mataga equation given below [19,20]

(1) $$\Delta \nu =\frac{2\Delta f}{4\pi \varepsilon_{0}ћca^{3}}\;{\left(\mu_{e}-\;\mu_{g}\right)}^{2}+\;b$$

(2) $$\Delta f\;=\frac{\varepsilon -1}{2\varepsilon +1}-\frac{n^{2}-1}{2n^{2\;}+1}$$

in which Δ*ν* = *ν_abs_* − *ν_em_* stands for Stokes shift, *ν_abs_* and *ν_em_* are absorption and emission frequency (cm^−1^), ħ is the Planck’s constant, *c* is the velocity of light in vacuum, *a* is the Onsager radius, and *b* is a constant. Δ*f* is the orientation polarizability, *ε* is the refractive index, *n* is the dielectric constant, *μ_e_* and *μ_g_* are the dipole moments of the emissive and ground states, respectively, and *ε*_0_ is the permittivity of the vacuum. (*μ_e_* − *μ_g_*)^2^ is proportional to the slope of the Lippert-Mataga plot.

**Figure 5:**
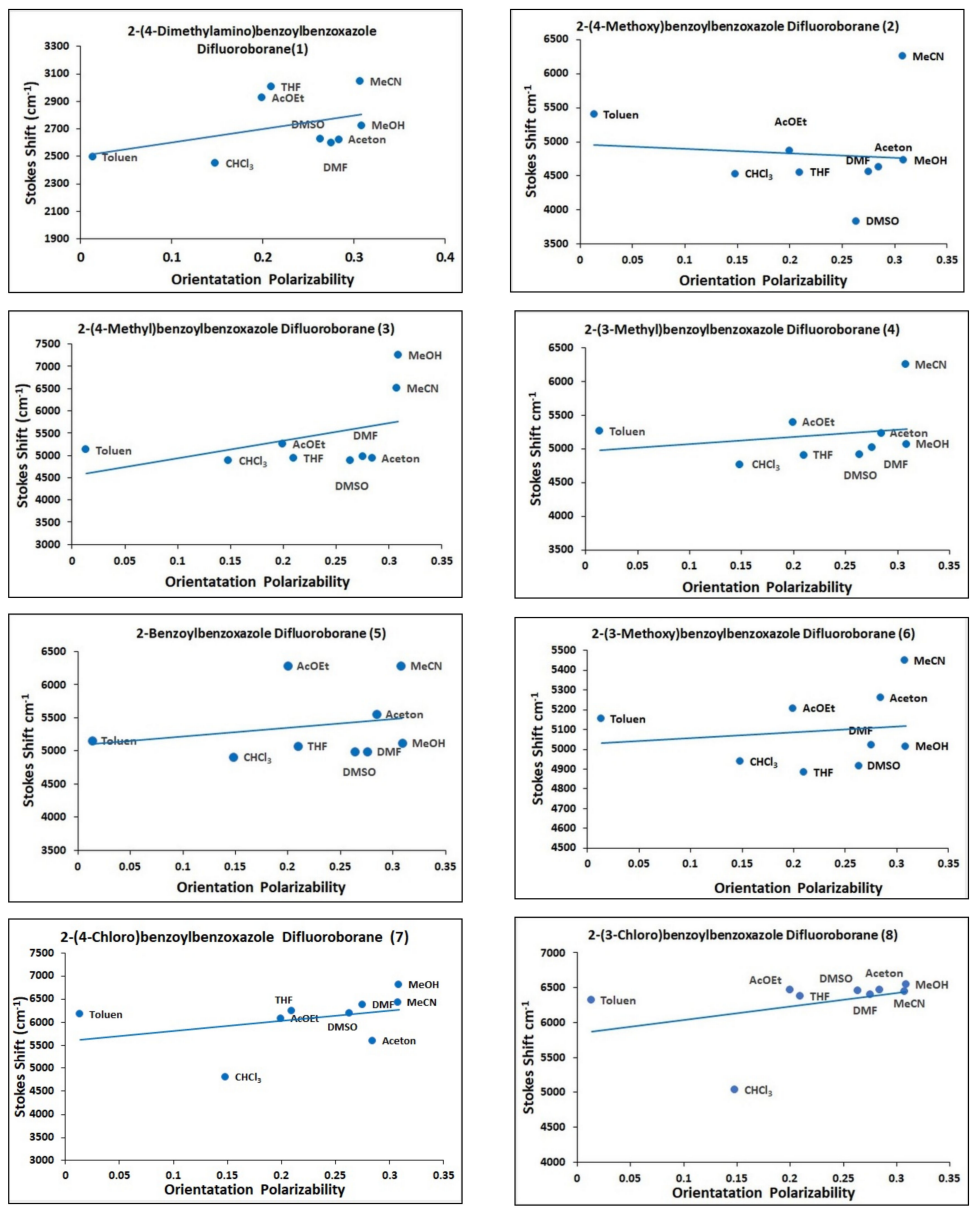
Lippert–Mataga plot of compounds **1**–**8**.

Lippert-Mataga plots showed higher Stokes shifts for all compounds in acetonitrile as a solvent, nonlinear nature of the plot was observed in acetonitrile for **1**–**6** shown in Figure 5, while such behavior for **7** and **8** was observed in chloroform. Compound **7** exhibited similar nature to that of **8**.

### 2.4. Computational Details

**Figure 6:**
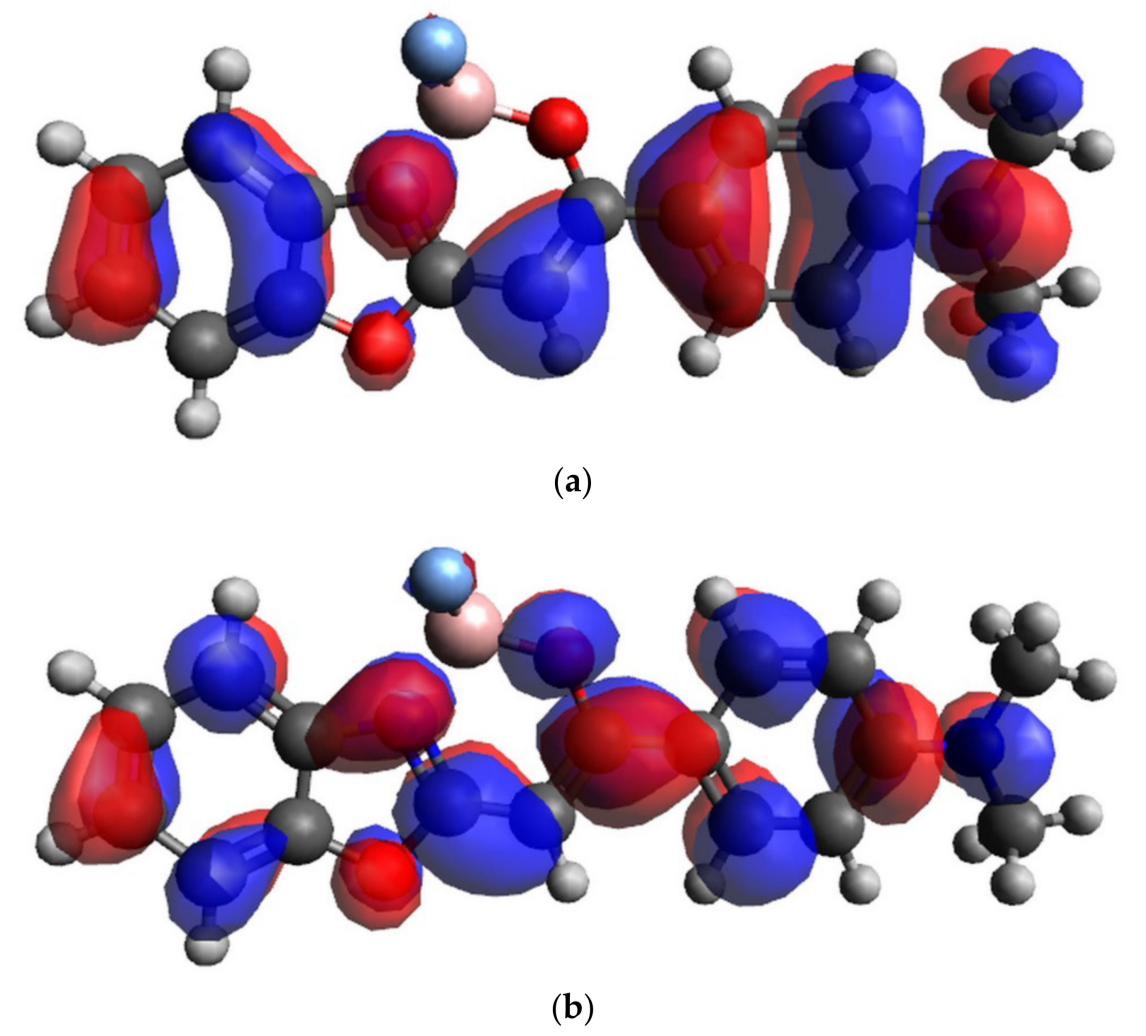
Distribution of HOMO (**a**) and LUMO (**b**) orbitals for 2-(4-dimethylamino)benzoyl-benzoxazole difluoroborane (**1**).

An important factor in assessing the practical use of the compounds under consideration is their reactivity. The use of computational chemistry methods allows us to describe this property of the tested molecules in the context of a set of descriptors based on the energy values of the frontal orbitals [21]. In Table 4, there are presented values characterizing all considered substituted 2-phenacylbenzoxazole difluoroboranes, including energy values of HOMO and LUMO orbitals, energy gaps, and hardness (η). Obtained data show that among all considered molecules, the highest reactivity is exhibited by the derivative containing 4-dimethylamino substituent. Such a molecule is characterized by the lowest values of hardness and energy gap. The distribution of HOMO and LUMO orbitals obtained for this molecule is presented in Figure 6. The rest of the considered derivatives exhibit quite similar values of both reactivity descriptors, however, there is observed a trend indicating that substitution in the fourth position to aromatic ring is more favorable than in the third. Taking into account all considered substituents, a decrease in reactivity is observed in the following order 4-NMe_2_ > 4-OMe > 4-Cl > 4-Me > 3-Cl > H > 3-Me > 3-OMe.

### 2.5. Vibrational Analysis

**Figure 7:**
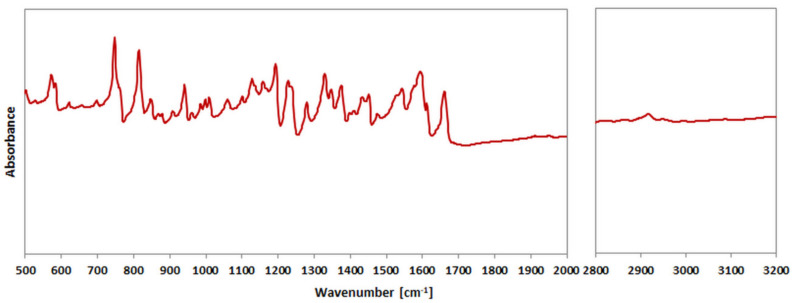
The observed IR absorption spectra of 2-(4-dimethylamino)benzoylbenzoxazole (**1b**) in the region 500–2000 cm^−1^ and 2800–3200 cm^−1^.

Vibrational spectroscopy is used extensively for the study of molecular conformations, identification of functional groups and reaction kinetics, etc. Spectroscopy FT-IR and quantum chemical calculations using the B3LYP functional and 6-311++G(d,p) basis set have been employed to study the structure of the title compounds. Substituted 2-phenacylbenzoxazoles **1b**–**8b** (starting compounds that were converted to BF_2_ complexes) exist in two tautomeric forms, the ketimine form (**K**) and enolimine form (**O**), through keto-enol tautomerization. A strong band observed in the FT-IR spectrum at around 1660 cm^−^^1^ was assigned to the C=O stretching vibration of ketimine tautomer. The free hydroxyl group absorbs strongly in the region 3700–3584 cm^−^^1^, whereas the existence of intramolecular hydrogen bond formation can lower the O–H stretching frequency in the range 3500–3200 cm^−^^1^ with an increase in intensity and breadth [22]. In the present study, a strong band observed at about 3380 cm^−^^1^ in FT-IR spectrum was assigned to O–H stretching vibration. The observed FT-IR spectrum of 2-(4-dimethylamino)benzoylbenzoxazole (**1b**), where the intensity was plotted against the vibrational wavenumber, is shown in Figure 7.

**Figure 8:**
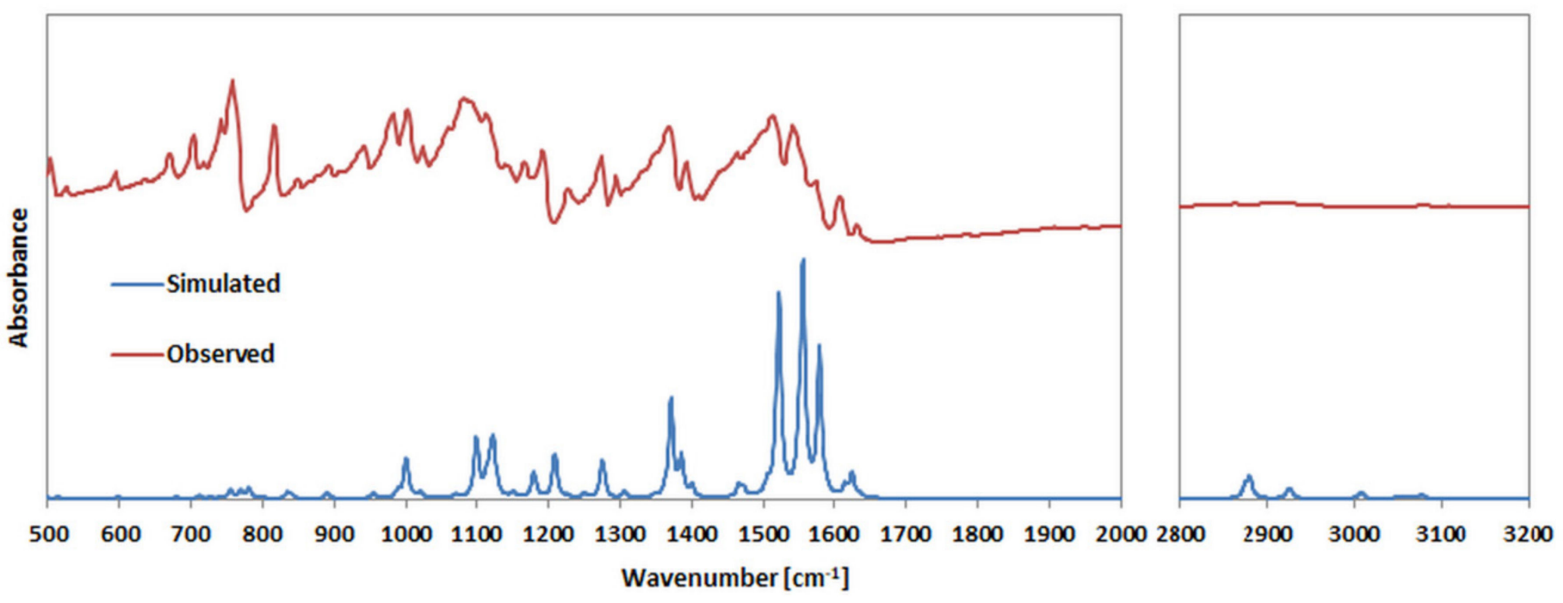
Comparison of the observed with simulated (scaled) IR absorption spectra of 2-(4-dimethylamino)benzoylbenzoxazole difluoroborane (**1**) in the region 500–2000 cm^−1^ and 2800–3200 cm^−1^.

For the substituted 2-phenacylbenzoxazole difluoroboranes **1**–**8**, no O–H and C=O vibrations could be observed in the FT-IR spectra. The theoretical spectrogram for the FT-IR spectrum of 2-(4-dimethylamino)benzoylbenzoxazole difluoroborane (**1**) was also constructed and compared with the experimental spectrum. The computed vibrational frequencies of this molecule were found in good agreement with experimental results. To improve the numerical agreement, the linear scaling method as given by Yoshida et al. [23] has been used. In this, the calculated harmonic vibrational wavenumbers have been scaled by the formula (ν_obs_/ν_cal_ = (1.0087–0.000016 × ν_cal_) cm^−1^) to facilitate a better agreement with the observed values. The value of the correlation coefficient was found to be R^2^ = 0.9997, which shows good agreement of the simulated wavenumbers with the observed one for the molecule of 2-(4-dimethylamino)benzoylbenzoxazole difluoroborane (**1**). The comparison of the experimental and simulated IR spectrum is presented in Figure 8.

## 3. Experimental

### 3.1. Materials

All reagents and solvents were purchased from Sigma-Aldrich (Poznań, Poland) and used without further purification. The highest (≥99%) purity of all used chemicals was required for spectroscopic studies.

### 3.2. Synthesis

The compounds were obtained from 2-phenacylbenzoxazoles **1b**–**8b** (Scheme 2) as described earlier [15]. The typical procedure was as follows: BF_3_ etherate (three equivalents) was added to the magnetically stirred solution (nitrogen atmosphere) of substituted 2-phenacylbenzoxazole (1 g) in dry chloroform (15–20 mL) and *N*-ethyldiisopropylamine (three equivalents). Then the solution was stirred overnight at room temperature, and concentrated Na_2_CO_3_ water solution (20 mL) was added slowly to the mixture. The organic fraction was separated, the water layer extracted with chloroform (two times using ca. 20–30 mL), dried (Na_2_SO_4_) and evaporated under reduced pressure. Residual solids were purified by flash chromatography (SiO_2_) using DCM as an eluent.

#### Elemental Analysis Is as Follows

*2-(4-Dimethylamino)benzoylbenzoxazole Difluoroborane* (**1**) Orange solid, yield 41%, m.p. 298–300 °C. ^1^H-NMR (CDCl_3_ from TMS) δ (ppm): 7.96 (d, 2H, ^3^*J*_H_,_H_ = 9.12 Hz), 7.85 (d, 1H, ^3^*J*_H_,_H_ = 7.36 Hz), 7.61 (d, 1H, ^3^*J*_H_,_H_ = 7.64 Hz), 7.50 (m, 2H), 6.89 (s, 1H), 6.81 (d, 2H, ^3^*J*_H_,_H_ = 9.16 Hz), 3.07 (s, 6H). ^11^B-NMR (CDCl_3_ from BF_3_⋅Et_2_O) δ (ppm): 1.79, (t). ^13^C-NMR δ (ppm): 172.1, 153.7, 152.4, 148.2, 130.6, 129.6, 127.0, 125.9, 119.1, 114.2, 112.1, 111.8, 77.7, ca. 40.61–39.35 (overlapped with solvent). ^15^N-NMR (CDCl_3_ from MeNO_2_) δ (ppm): −313.97. ^19^F-NMR (CDCl_3_ from CFCl_3_) δ (ppm): −134.64. C_17_H_15_BF_2_N_2_O_2_, Calcd. C, 62.23; H, 4.61; N, 8.54. Found C, 62.14; H, 4.64; N, 8.39.

*2-(4-Methoxy)benzoylbenzoxazole Difluoroborane* (**2**) Yellow solid, yield 39%, m.p. 242–243 °C (251–253 °C [9]). ^1^H-NMR (CDCl_3_ from TMS) δ (ppm): 7.98 (m, 2H), 7.78 (d, 1H, ^3^*J*_H_,_H_ = 7.84 Hz), 7.55 (d, 1H, ^3^*J*_H_,_H_ = 8.16 Hz), 7.47 (m, 1H), 7.40 (m, 1H), 6.98 (m, 2H), 6.37 (s, 1H), 3.89 (s, 3H). ^11^B-NMR (CDCl_3_ from BF_3_⋅Et_2_O) δ (ppm): 1.80, (t). ^13^C-NMR δ (ppm): 172.2 164.9, 163.3, 148.1, 130.6, 129.3, 126.6 125.6, 115.2, 114.1, 111.0, 78.8, 55.5. ^15^N-NMR (CDCl_3_ from MeNO_2_) δ (ppm): −215.72. ^19^F-NMR (CDCl_3_ from CFCl_3_) δ (ppm): −135.59. C_16_H_12_BF_2_NO_3_, Calcd. C, 60.99; H, 3.84; N, 4.45. Found C, 60.69; H, 3.99; N, 4.60.

*2-(4-Methyl)benzoylbenzoxazole Difluoroborane* (**3**) Yellow solid, yield 47%, m.p. 256–258 °C. ^1^H-NMR (CDCl_3_ from TMS) δ (ppm): 7.91 (m, 2H), 7.80 (d, 1H, ^3^*J*_H_,_H_ = 8.36 Hz), 7.57 (m, 1H), 7.48 (m, 1H), 7.40 (m, 1H), 7.41 (m, 1H), 7.29 (d, 2H, ^3^*J*_H_,_H_ = 8.00 Hz), 6.44 (s, 1H) 2.44 (s, 3H). ^11^B-NMR (CDCl_3_ from BF_3_⋅Et_2_O) δ (ppm): 1.84, (t). ^13^C-NMR δ (ppm): 172.6, 164.9, 148.2, 143.5, 130.6, 129.5, 127.3, 126.7, 125.8, 115.4, 111.1, 79.7, 21.7. ^15^N-NMR (CDCl_3_ from MeNO_2_) δ (ppm): −215.01. ^19^F-NMR (CDCl_3_ from CFCl_3_) δ (ppm): −135.34. C_16_H_12_BF_2_NO_2_, Calcd. C, 64.25; H, 4.04; N, 4.68. Found C, 64.35; H, 3.94; N, 4.68.

*2-(3-Methyl)benzoylbenzoxazole Difluoroborane* (**4**) Yellow solid, yield 48%, m.p. 230–231 °C. ^1^H-NMR (CDCl_3_ from TMS) δ (ppm): 7.85 (s, 1H), 7.76 (m, 2H), 7.57 (m, 1H), 7.49 (m, 1H), 7.49 (m, 1H), 7.42 (m, 1H), 7.37 (m, 2H), 6.47 (s, 1H) 2.44 (s, 3H). ^11^B-NMR (CDCl_3_ from BF_3_⋅Et_2_O) δ (ppm): 1.86, (t). ^13^C-NMR δ (ppm): 172.7, 164.8, 148.2, 138.6, 133.4, 133.1, 130.5, 128.6, 127.9, 126.7, 125.9, 124.4, 115.4, 111.1, 80.3, 21.4. ^15^N-NMR (DMSO-*d_6_* from MeNO_2_) δ (ppm): −214.18. ^19^F-NMR (CDCl_3_ from CFCl_3_) δ (ppm): −135.14. C_16_H_12_BF_2_NO_2_, Calcd. C, 64.25; H, 4.04; N, 4.68. Found C, 63.92; H, 4.1; N, 4.85.

*2-Benzoylbenzoxazole Difluoroborane* (**5**) Yellowish-green solid, yield 38%, m.p. 239.2–240.8 °C (244–246 °C [9]). ^1^H-NMR (CDCl_3_ from TMS) δ: 8.02 (m, 2H), 7.82 (m, 1H), 7.57 (m, 2H) 7.51 (m, 2H), 7.45 (m, 2H), 6.49 (s, 1H). ^11^B-NMR (CDCl_3_ from BF_3_⋅Et_2_O) δ: 1.85, (t). ^13^C-NMR δ: 170.8, 165.1, 148.5, 133.1, 130.1, 129.5, 127.5, 126.9, 126.4, 114.9, 112.5, 82.1. ^15^N-NMR (CDCl_3_ from MeNO_2_) δ: −213.54. ^19^F-NMR (CDCl_3_ from CFCl_3_) δ: −135.07. C_15_H_10_BF_2_NO_2_, Calcd. C, 63.20; H, 3.54; N, 4.91. Found C, 63.25; H, 3.49; N, 4.83.

*2-(3-Methoxy)benzoylbenzoxazole Difluoroborane (***6***) Yellow solid, yield 42%,* m.p. 218–220 °C (235–237 °C [9]). ^1^H-NMR (CDCl_3_ from TMS) δ (ppm): 7.82 (d, 1H, ^3^*J*_H_,_H_ = 7.84 Hz), 7.56 (m, 3H), 7.49 (m, 1H), 7.44 (m, 1H), 7.40 (m, 1H), 7.09 (m, 1H), 6.47 (s, 1H) 3.9 (s, 3H). ^11^B-NMR (CDCl_3_ from BF_3_⋅Et_2_O) δ (ppm): 1.84, (t). ^13^C-NMR δ (ppm): 172.3, 164.7, 159.9, 148.2, 134.6, 130.5, 129.7, 126.8, 126.0, 119.6, 118.9, 115.5, 112.0, 111.2, 80.7, 55.6. ^15^N-NMR (CDCl_3_ from MeNO_2_) δ (ppm): −213.97. ^19^F-NMR (CDCl_3_ from CFCl_3_) δ (ppm): −134.98. C_16_H_12_BF_2_NO_3_, Calcd. C, 60.99; H, 3.84; N, 4.45. Found C, 60.72; H, 4.01; N, 4.95.

*2-(4-Chloro)benzoylbenzoxazole Difluoroborane* (**7**) Yellow solid, yield 37%, m.p. 233–235 °C (250 °C [10]). ^1^H-NMR (CDCl_3_ from TMS) δ (ppm): 7.94 (m, 2H), 7.82 (d, 1H, ^3^*J*_H_,_H_ = 7.84 Hz), 7.58 (m, 1H), 7.51 (m, 1H), 7.47 (m, 2H), 7.44 (m, 1H), 6.45 (s, 1H). ^11^B-NMR (CDCl_3_ from BF_3_⋅Et_2_O) δ (ppm): 1.81, (t). ^13^C-NMR δ (ppm): 171.0, 164.5, 148.2, 138.8, 131.6, 130.4, 129.1, 128.5, 126.9, 126.2, 115.5, 111.2, 80.6. ^15^N-NMR (CDCl_3_ from MeNO_2_) δ (ppm): −213.00. ^19^F-NMR (CDCl_3_ from CFCl_3_) δ (ppm): −134.98. C_15_H_9_BF_2_NO_2_, Calcd. C, 56.39; H, 2.84; N, 4.38. Found C, 56.51; H, 2.75; N, 4.41.

*2-(3-Chloro)benzoylbenzoxazole Difluoroborane* (**8**) Yellow solid, yield 34%, m.p. 228–230 °C. ^1^H-NMR (CDCl_3_ from TMS) δ (ppm): 8.00 (t, 1H), 7.88 (m, 1H), 7.83 (d, 1H, ^3^J_H_,_H_ = 7.84 Hz), 7.60 (m, 1H), 7.52 (m, 2H), 7.45 (m, 2H), 6.48 (s, 1H). ^11^B-NMR (CDCl_3_ from BF_3_⋅Et_2_O) δ (ppm): 1.81, (t). ^13^C-NMR δ (ppm): 170.6, 164.4, 148.3, 135.1, 135.0, 132.4, 130.4, 130.1, 127.3, 126.9, 126.3, 125.3, 115.6, 111.3, 81.2. ^15^N-NMR (CDCl_3_ from MeNO_2_) δ (ppm): −212.43. ^19^F-NMR (CDCl_3_ from CFCl_3_) δ (ppm): −134.78. C_15_H_9_BF_2_NO_2_, Calcd. C, 56.39; H, 2.84; N, 4.38. Found C, 56.26; H, 2.81; N, 4.54.

### 3.3. Measurements

The ^1^H-NMR spectra were recorded using an Ascend III spectrometer operating at 400 MHz, Bruker (Bydgoszcz, Poland). Chloroform was used as a solvent and tetramethylsilane (TMS) as the internal standard. Chemical shifts (δ) were reported in ppm relative to TMS and coupling constants (J) in Hz.

The elemental analysis was made with a Vario MACRO 11.45–0000, Elemental Analyser System GmbH, operating with the VARIOEL software (version 5.14.4.22).

The melting point was measured on the Melting Point M-565 Apparatus (Buchi) with the measuring speed 5 °C/min.

The absorption and emission spectra were measured at room temperature in a quartz cuvette (1 cm) using an Agilent Technology UV-Vis Cary 60 Spectrophotometer and a Hitachi F-7000 Spectrofluorometer, respectively.

The fluorescence quantum yields for the compounds in chloroform were determined as follows, the fluorescence spectrum of diluted (A ≈ 0.1) boranes solution was recorded by excitation at the absorption band maximum of the reference. Diluted 9,10-diphenylanthracene in cyclohexane (*ϕ* = 0.93) [24] was used as reference. The fluorescence spectrum of 9,10-diphenylanthracene was obtained by excitation at its absorption peak at 355 nm. The quantum yield of the tested compounds (*ϕ_dye_*) was calculated using the following equation [25]:

(3) $$\phi_{dye}=\phi_{ref}\;\cdot \;\frac{I_{dye}}{I_{ref}}\frac{A_{ref}}{A_{dye}}\;\cdot \;\frac{n_{dye}^{\;2}}{n_{ref}^{\;2}}$$

where *ϕ_ref_* is the fluorescence quantum yield of the reference sample (9,10-diphenylanthracene) in cyclohexane, *A_dye_* and *A_ref_* are the absorbance of the dye and reference samples at the excitation wavelengths (355 nm), *I_dye_* and *I_ref_* are the integrated emission intensity for the dyes and references sample, *n_dye_* and *n_ref_* are the refractive indices of the solvents used for the dyes and reference, respectively. Coumarine 153 in cyclohexane (*ϕ* = 0.90 [26]; *λ_ex_* = 393 nm) was used as reference standard for compound carrying −NMe_2_ group.

Infrared spectra have been recorded in the region 360–7000 cm^−^^1^ on a Bruker Alpha FT-IR spectrometer with a spectral resolution of 2 cm^−^^1^ using the ATR (attenuated total reflectance) method.

The geometry optimization, vibrational spectra, and the HOMO and LUMO energies were calculated based on density functional theory with the use of B3LYP [27,28,29] functional and 6-311++G(d,p) basis set [30,31]. All calculations were carried out using Gaussian 09 software [32]. The analysis of the frontier orbitals and IR spectra including extraction of frequencies and a visual presentation of the vibrational modes and orbitals was realized with the use of Avogadro 1.2.0 application [33].

## 4. Conclusions

The indicated aims were achieved through a synthesis of a series of 2-phenacylbenzoxazole difluoroboranes substituted by a weaker electron-withdrawing (Cl) and electron-releasing (4-Me, 4-OMe, and 4-NMe*_2_*) substituents. Complexes have been identified based on a magnetic atomic nucleus ^1^H, ^11^B, ^13^C, ^15^N, and ^19^F isotope resonance spectra. ^15^N-NMR shifts correlate with the substituent character of the phenyl ring with R^2^ = 0.91. The spectral data show that the absorption and fluorescence properties of 2-phenacylbenzoxazole difluoroboranes **1**–**8** depend on the character of the substituent. The experimental results indicate the positive solvatochromism of studied compounds with increasing solvent polarity. Among all derivatives, only 2-(4-dimethylamino)-benzoylbenzoxazole difluoroborane **1** (the NMe_2_ group was the strongest electron donor in the series) presented strong and relatively red-shifted fluorescence in the chloroform. Other complexes hardly demonstrated any fluorescence, indicating that probably only benzoxazole-boron complexes with strongly electron-releasing groups like amino groups could be attractive for their use as new fluorescent molecules for future applications in bio-labeling, medicine, or fluorescence microscopy, and they might be alternative or supplementary to popular BODIPY dyes. Furthermore, the study using these compounds as photosensitizers in the photopolymerization process is still in progress.

## Figures and Tables

**Table 1:** Selected ^1^H, ^11^B, ^13^C, ^15^N and ^19^F-NMR chemical shifts of 2-phenacylbenzoxazole difluoroboranes **1**–**8** for 0.1–0.2 M solution in CDCl_3_ at 30 °C.

No.	Substituent	C10	C11	H10	B13	F13	N13
**1**	4-NMe_2_	77.74	172.13	6.81	1.79	−134.64	-
**2**	4-OMe	78.85	172.23	6.37	1.80	−135.59	−215.72
**3**	4-Me	79.74	172.63	6.44	1.84	−135.34	−215.01
**4**	3-Me	80.33	172.70	6.47	1.85	−135.14	−214.18
**5**	H	82.11	170.79	6.49	1.85	−135.07	−213.54
**6**	3-OMe	80.56	172.28	6.47	1.84	−134.98	−213.97
**7**	4-Cl	80.63	171.01	6.45	1.81	−134.98	−213.00
**8**	3-Cl	81.17	170.64	6.48	1.81	−134.78	−212.43

**Table 2:** Photophysical properties of examined compounds measured in chloroform.

No.	Substituent	λ_abs_ (nm)	λ_fl_ (nm)	ε (M^−1^·cm^−1^)	Stokes Shift (cm^−1^)	ϕ_fl_ (× 10^−2^)
**1**	4-NMe_2_	410	458	42,500	2449	98.18
**2**	4-OMe	362	433	32,900	4530	1.82
**3**	4-Me	354	428	31,219	4884	0.66
**4**	3-Me	352	423	22,961	4769	0.48
**5**	H	350	424	27,000	4905	0.54
**6**	3-OMe	354	429	26,727	4938	0.57
**7**	4-Cl	355	428	29,615	4805	0.58
**8**	3-Cl	353	430	29,648	5073	0.50

**Table 3:** Spectroscopic properties of 2-phenacylbenzoxazole difluoroboranes **1**–**8** in solvents of different polarity.

No.		Toluene	CHCl_3_	THF	AcOEt	Acetone	MeOH	DMF	MeCN	DMSO
**1**	λ_abs_ (nm)	407	410	408	407	420	419	426	415	431
	λ_flu_ (nm)	453	458	465	462	427	473	479	475	486
	Stokes (cm^−1^)	2495	2449	3004	2925	2623	2725	2597	3043	2626
**2**	λ_abs_ (nm)	362	362	361	359	360	358	363	359	375
	λ_flu_ (nm)	450	433	432	435	432	431	435	463	438
	Stokes (cm^−1)^	5402	4530	4553	4867	4630	4731	4560	6257	3836
**3**	λ_abs_ (nm)	355	354	354	352	352	351	355	351	356
	λ_flu_ (nm)	434	428	429	432	426	471	431	455	431
	Stokes (cm^−1^)	5127	4884	4938	5261	4935	7259	4967	6512	4888
**4**	λ_abs_ (nm)	354	352	353	351	351	349	353	350	355
	λ_flu_ (nm)	435	423	427	433	430	424	429	448	430
	Stokes (cm^−1^)	5261	4769	4909	5395	5234	5068	5019	6250	4913
**5**	λ_abs_ (nm)	354	350	351	349	349	349	352	349	354
	λ_flu_ (nm)	433	424	425	447	433	425	427	447	456
	Stokes (cm^−1^)	5154	4905	5071	6282	5559	5124	4990	6282	4993
**6**	λ_abs_ (nm)	356	354	354	352	352	351	355	351	357
	λ_flu_ (nm)	436	429	428	431	432	426	432	434	455
	Stokes (cm^−1^)	5154	4938	4884	5207	5261	5016	5021	5448	4916
**7**	λ_abs_ (nm)	357	355	355	352	353	352	355	352	358
	λ_flu_ (nm)	458	428	456	448	456	463	459	455	460
	Stokes (cm^−1^)	6431	4805	6239	6088	5601	6545	6382	6447	6462
**8**	λ_abs_ (nm)	354	353	352	351	351	350	353	350	354
	λ_flu_ (nm)	456	430	454	454	454	454	456	452	459
	Stokes (cm^−1^)	6319	5037	6383	6464	6464	6545	6399	6447	6462

**Table 4:** The values of hardness (η), energy gap, and energies of HOMO and LUMO orbitals were estimated for considered molecules.

No.	Substituent	HOMO (eV)	LUMO (eV)	Energy gap (eV)	η (eV)
**1**	4-NMe_2_	−5.664	−2.090	3.574	1.787
**2**	4-OMe	−6.168	−2.323	3.846	1.923
**3**	4-Me	−6.328	−2.423	3.906	1.953
**4**	3-Me	−6.389	−2.454	3.935	1.967
**5**	H	−9.438	−2.504	3.934	1.967
**6**	3-OMe	−6.390	−2.425	3.964	1.982
**7**	4-Cl	−6.523	−2.663	3.860	1.930
**8**	3-Cl	−6.595	−2.684	3.910	1.955

## Supplementary Materials

The following are available online for related NMR spectra.
