# Supplementary material for: Substituted 2-Phenacylbenzoxazole Difluoroboranes: Synthesis, Structure and Properties
Source: Molecules. 2020 Nov 19;25(22):5420. doi: 10.3390/molecules25225420 (PMC7699529; doi:10.3390/molecules25225420)
Supplement: Supplementary file 1 [file molecules-25-05420-s001.pdf]

Substituted 2-Phenacylbenzoxazole Difluoroboranes: Synthesis, Structure and Properties

Agnieszka Skotnicka*

Faculty of Chemical Technology and Engineering, UTP University of Science and Technology, Seminaryjna 3, 85-326, Bydgoszcz, Poland

*Correspondence should be addressed to Agnieszka Skotnicka: askot@utp.edu.pl

Supporting Information

Content

^{1}H NMR and ^{13}C NMR spectra of **1-8**

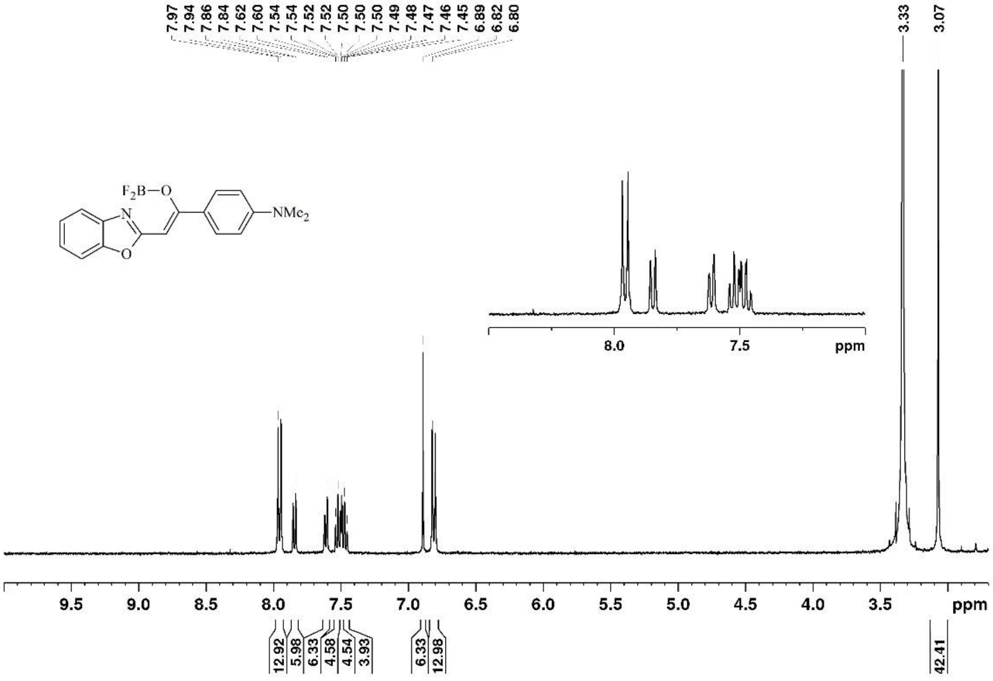

Figure S1. ^{1}H NMR spectrum (400 MHz) of **1** in $CDCl_3$.

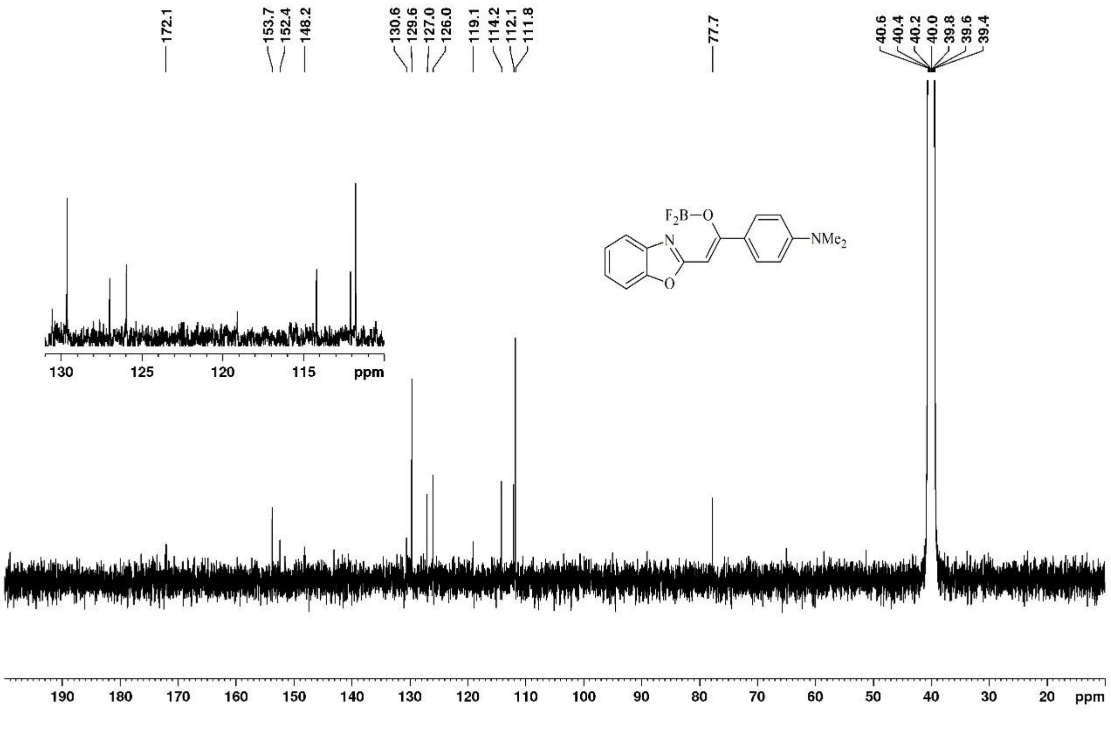

Figure S2. ^{13}C NMR spectrum (400 MHz) of **1** in $CDCl_3$.

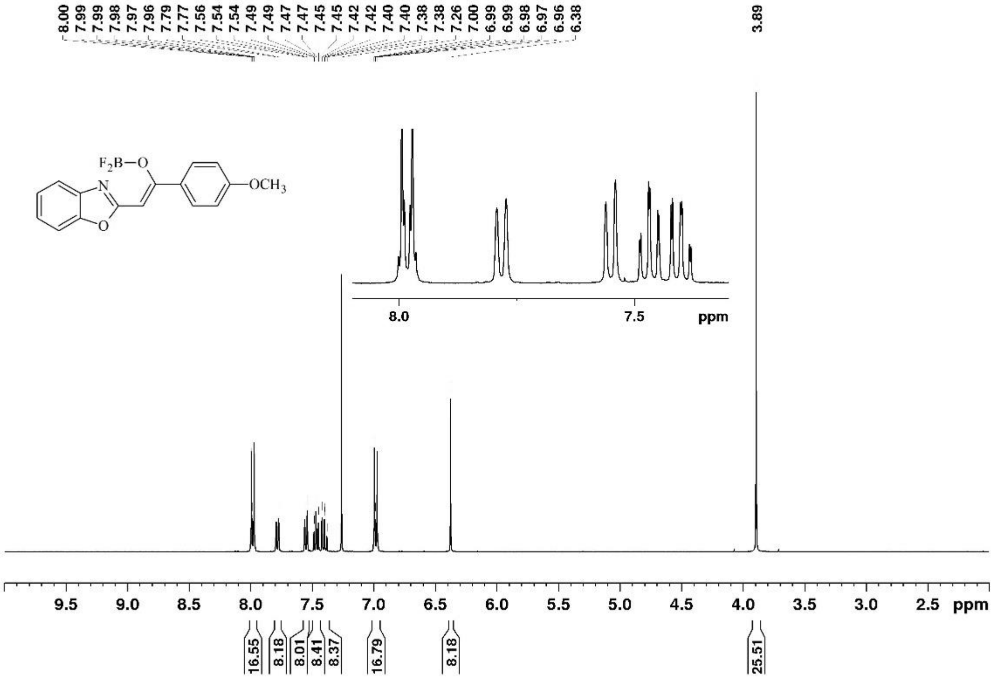

Figure S3. ^{1}H NMR spectrum (400 MHz) of **2** in $CDCl_3$.

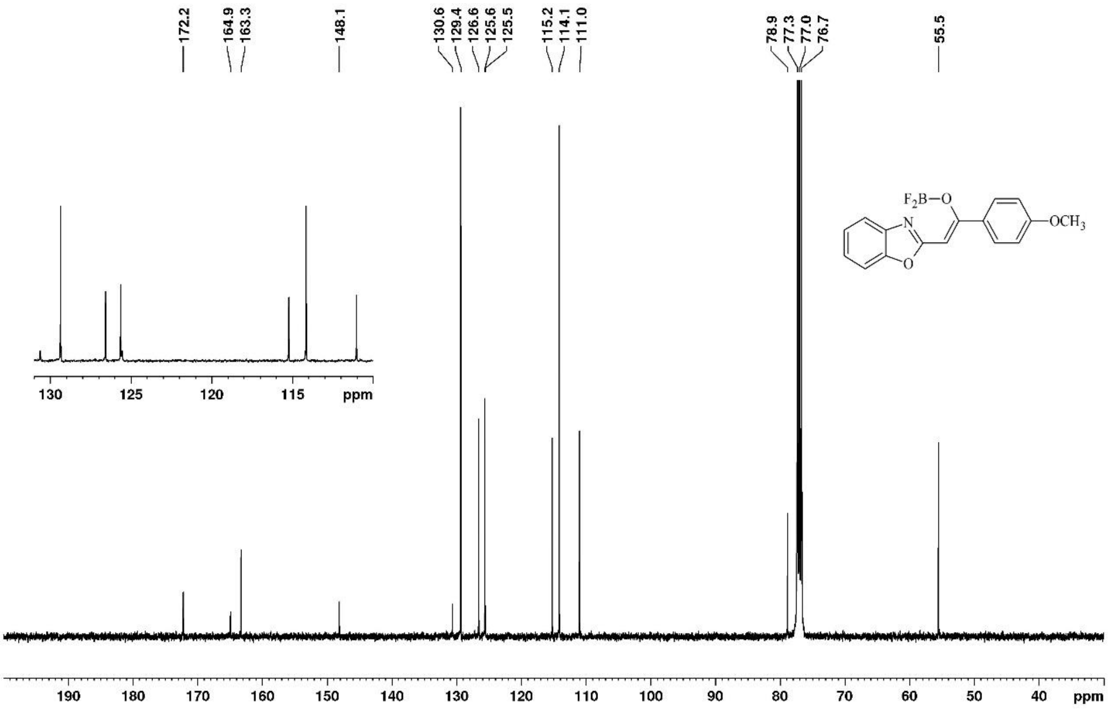

Figure S4. ^{13}C NMR spectrum (400 MHz) of **2** in $CDCl_3$.

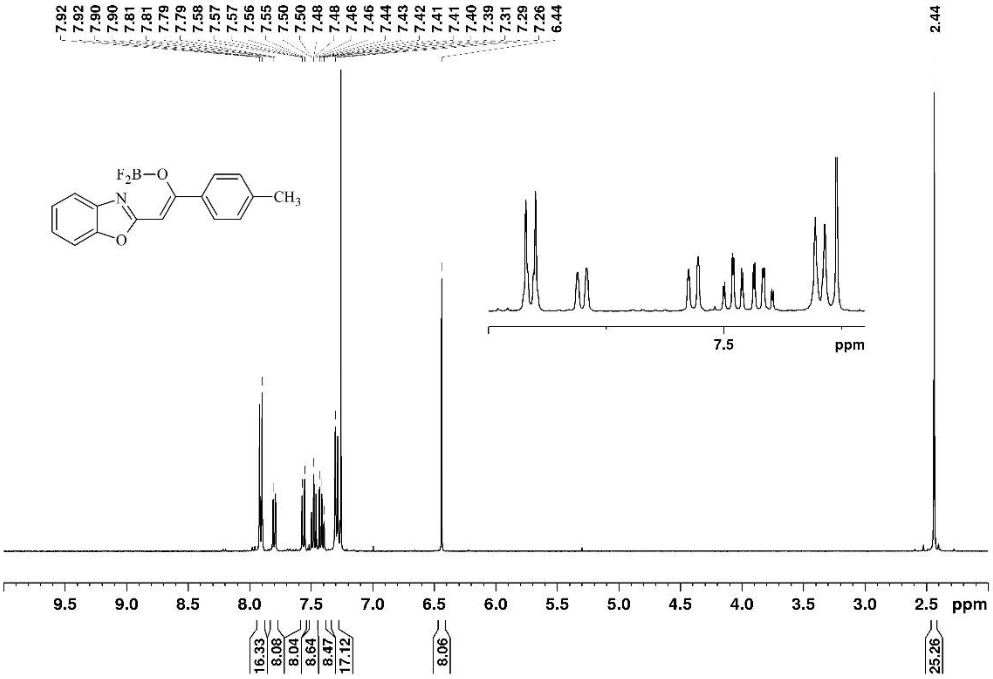

Figure S5. ^{1}H NMR spectrum (400 MHz) of **3** in $CDCl_3$.

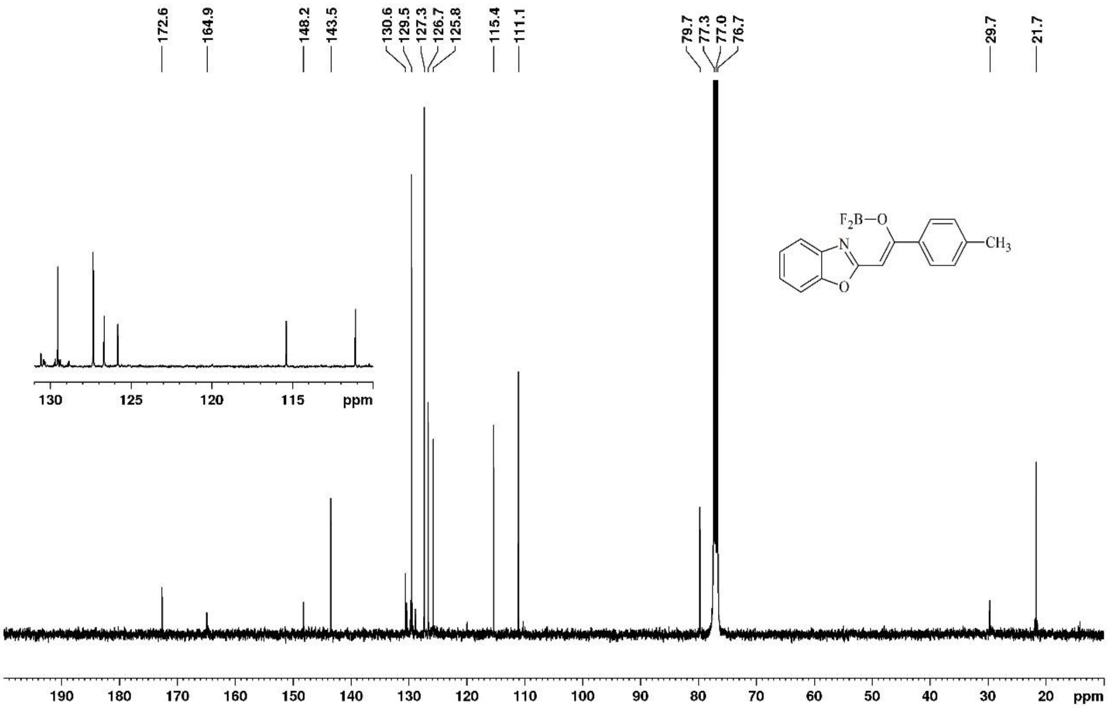

Figure S6. ^{13}C NMR spectrum (400 MHz) of **3** in $CDCl_3$.

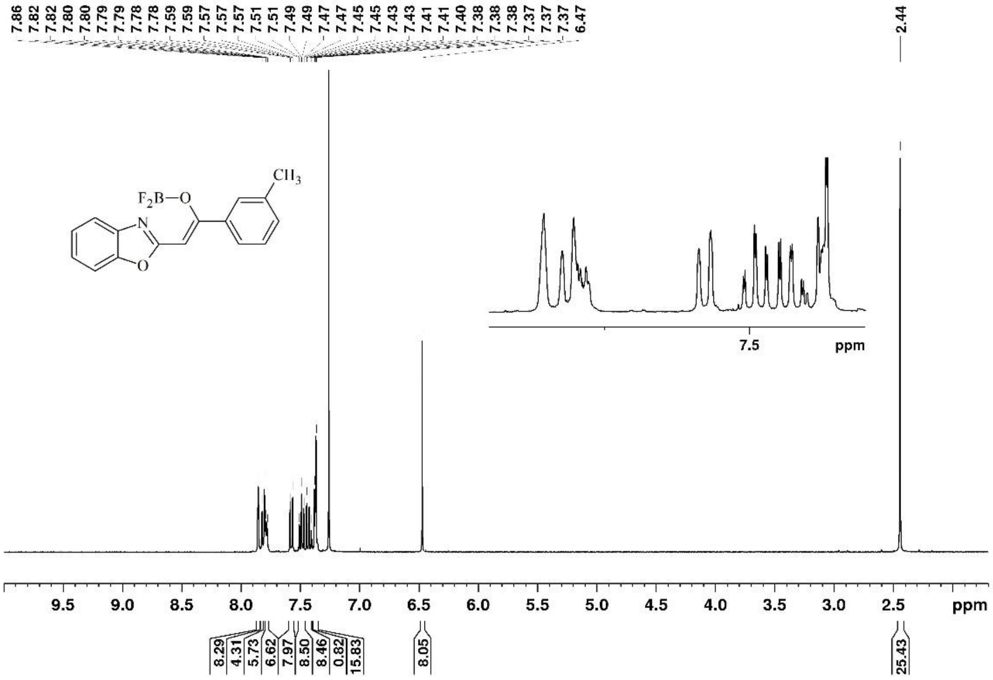

Figure S7. ^{1}H NMR spectrum (400 MHz) of **4** in $CDCl_3$.

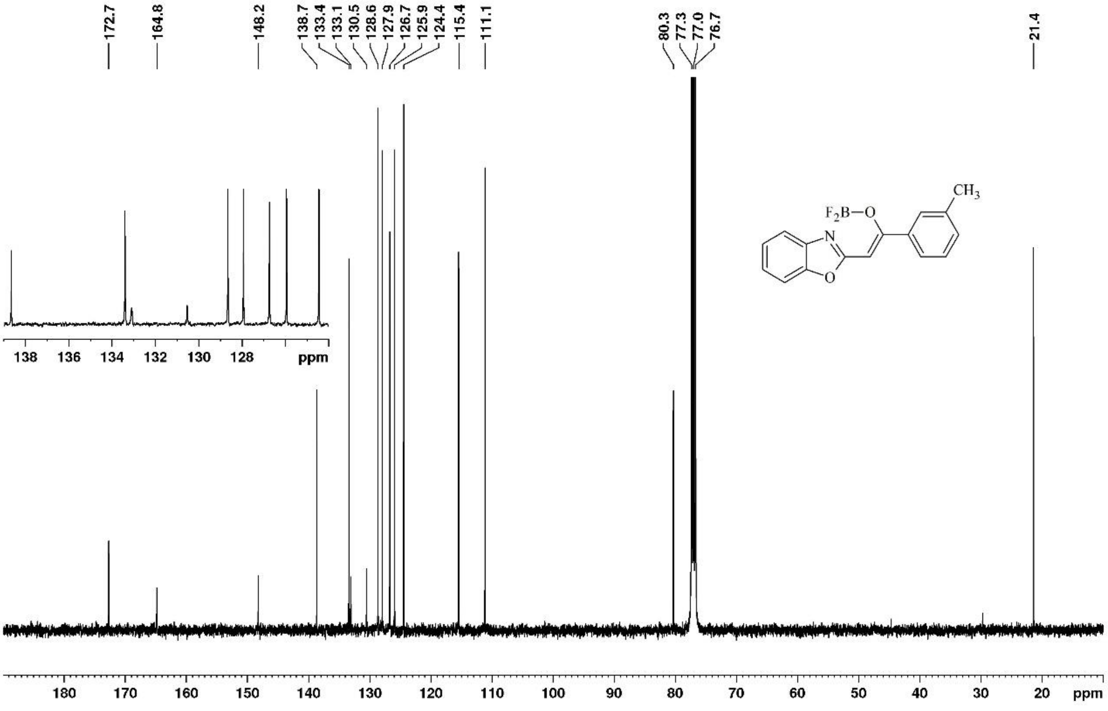

Figure S8. ^{13}C NMR spectrum (400 MHz) of **4** in $CDCl_3$.

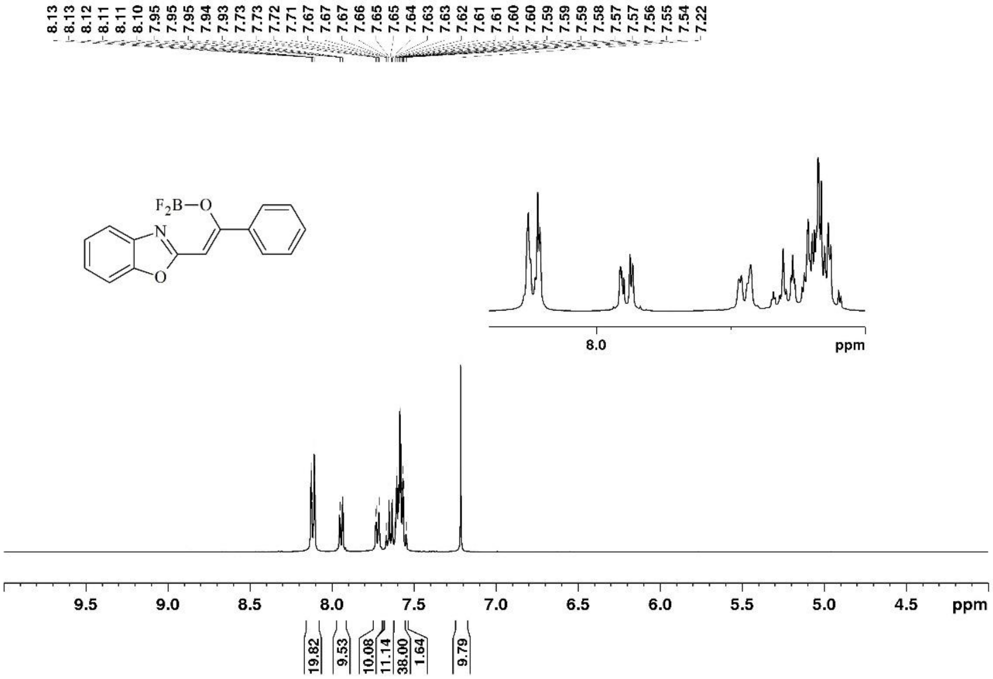

Figure S9. ^{1}H NMR spectrum (400 MHz) of **5** in $CDCl_3$.

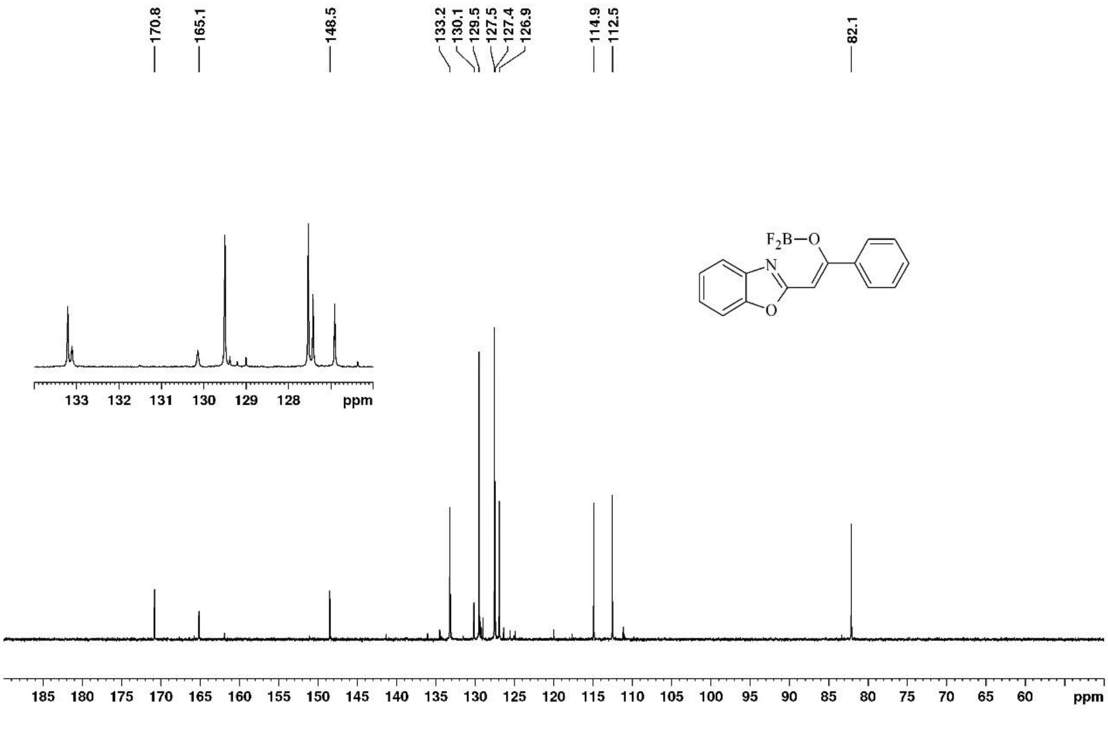

Figure S10. ^{13}C NMR spectrum (400 MHz) of **5** in $CDCl_3$.

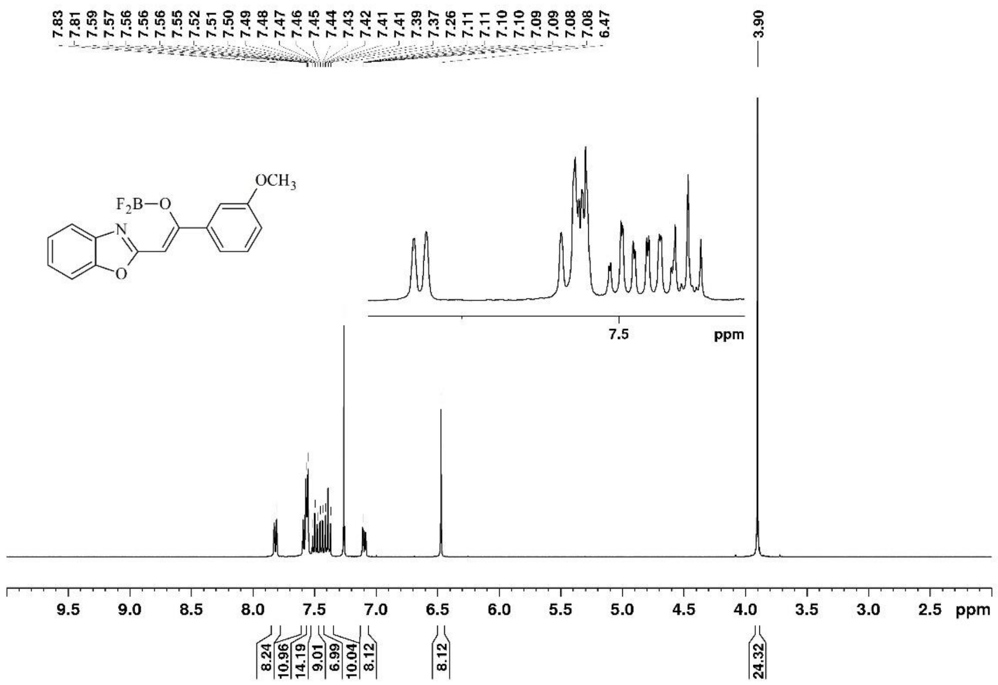

Figure S11. ^{1}H NMR spectrum (400 MHz) of **6** in $CDCl_3$.

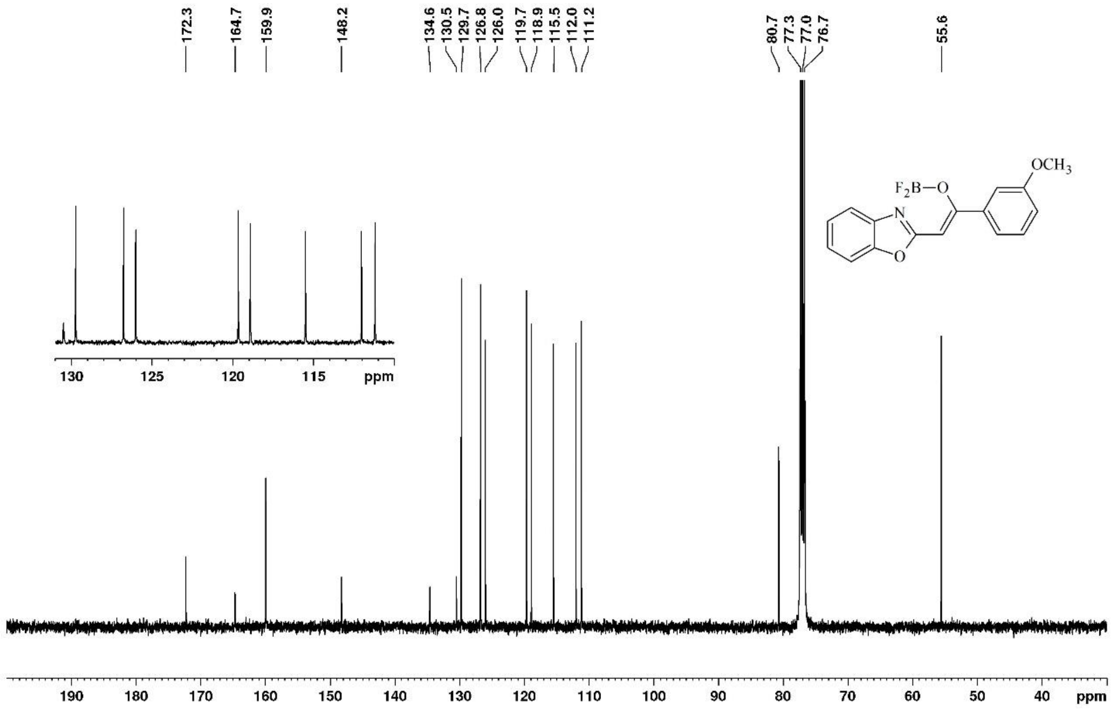

Figure S12. ^{13}C NMR spectrum (400 MHz) of **6** in $CDCl_3$.

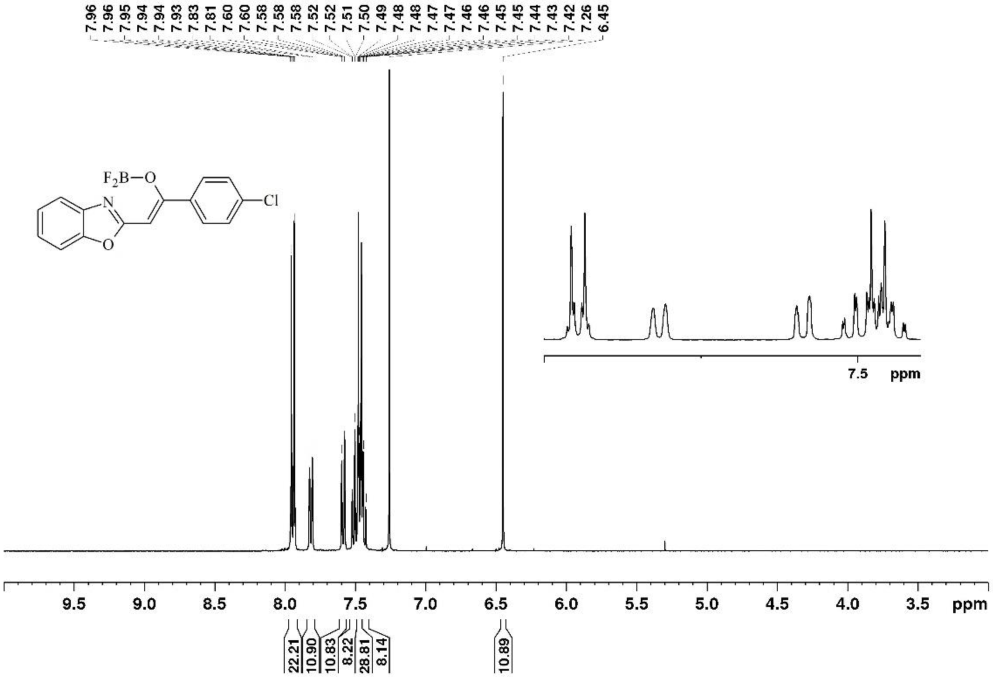

Figure S13. ^{1}H NMR spectrum (400 MHz) of **7** in $CDCl_3$.

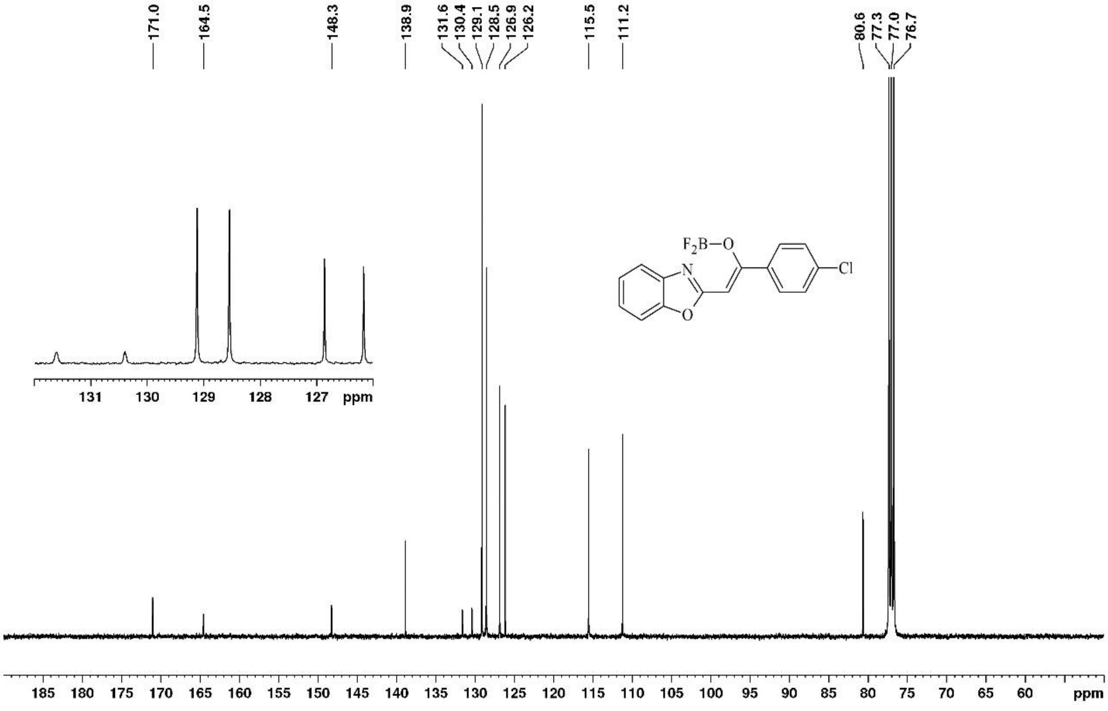

Figure S14. ^{13}C NMR spectrum (400 MHz) of **7** in $CDCl_3$.

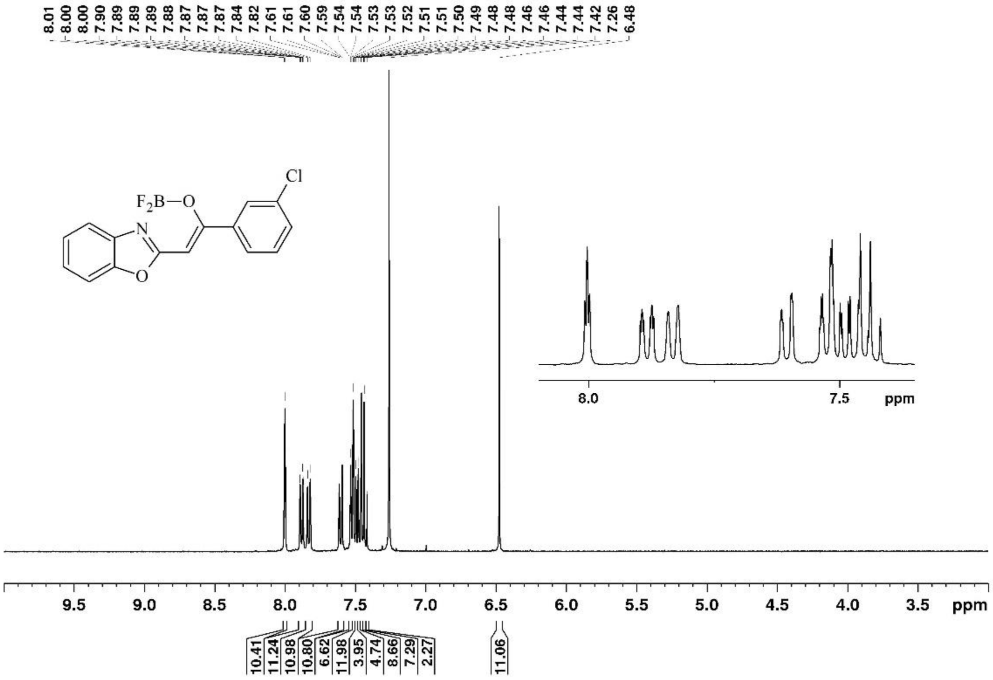

Figure S15. ^{1}H NMR spectrum (400 MHz) of **8** in $CDCl_3$.

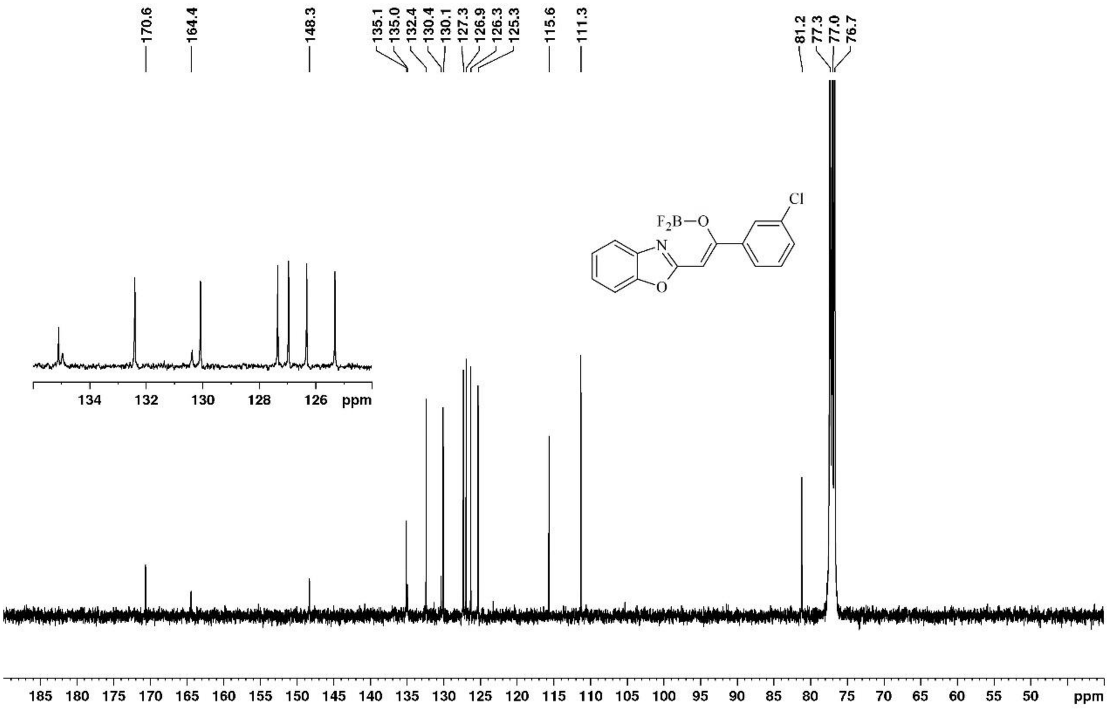

Figure S16. ^{13}C NMR spectrum (400 MHz) of **8** in $CDCl_3$
